# Beyond differential expression: the quest for causal mutations and effector molecules

**DOI:** 10.1186/1471-2164-13-356

**Published:** 2012-07-31

**Authors:** Nicholas J Hudson, Brian P Dalrymple, Antonio Reverter

**Affiliations:** 1 Computational and Systems BiologyCSIRO Livestock Industries, 306 Carmody Road St. Lucia, Brisbane, QLD 4067, Australia

**Keywords:** Differential connectivity, Differential networking, Gene expression, Causal mutation algorithm

## Abstract

High throughput gene expression technologies are a popular choice for researchers seeking molecular or systems-level explanations of biological phenomena. Nevertheless, there has been a groundswell of opinion that these approaches have not lived up to the hype because the interpretation of the data has lagged behind its generation. In our view a major problem has been an over-reliance on isolated lists of differentially expressed (DE) genes which – by simply comparing genes to themselves – have the pitfall of taking molecular information out of context. Numerous scientists have emphasised the need for better context. This can be achieved through holistic measurements of differential connectivity in addition to, or in replacement, of DE. However, many scientists continue to use isolated lists of DE genes as the major source of input data for common readily available analytical tools. Focussing this opinion article on our own research in skeletal muscle, we outline our resolutions to these problems – particularly a universally powerful way of quantifying differential connectivity. With a well designed experiment, it is now possible to use gene expression to identify causal mutations and the other major effector molecules with whom they cooperate, irrespective of whether they themselves are DE. We explain why, for various reasons, no other currently available experimental techniques or quantitative analyses are capable of reaching these conclusions.

## 

"“*Given one hour to solve a problem my life depended on, I would spend fifty five minutes defining the nature of the question to be solved. The remaining five minutes would be more than adequate to answer that question*.” Albert Einstein (1879–1955) on priorities in problem solving."

"“*An approximate answer to the right question is worth a great deal more than a precise answer to the wrong question*.” John Tukey (1915–2000) on priorities in problem solving."

"“*Low input, high throughput, no output!*” Sydney Brenner (1927-) on the application of high throughput gene expression technologies to biological problem solving."

In this correspondence piece we describe a previously published set of gene expression analyses [1-4] - developed on skeletal muscle, but universally applicable – that can correctly determine the site of causal mutations and prioritise major effector molecules, in the absence of Differential Expression (DE). Although the causal gene(s) may or may not be directly measurable through their own expression changes, they always cast a long transcriptional shadow over the rest of the data - and the shadow *can* be quantified.

This kind of mechanistic systems-level and molecular-level understanding surely underpins medical breakthroughs, productivity gains in agricultural commodities and many other diverse applications of biological modelling.

Gene expression analysis is a mathematical area, but we recognise that the interested end users may be practicing scientists without advanced mathematical training. Therefore, we have endeavoured to explain the mathematical approaches figuratively, making ample use of metaphor and analogy.

This piece is not an exhaustive historical review of gene expression network analyses. Readers interested in alternative examples of recent promising methods can be found here [5-8] and a recent review of differential networking can be found here [9]. Nor is this correspondence intended as a broader view on the various complementary data types and methods aimed at identifying causal genes and pathways such as expression QTL analyses, mutation enriched pathways [10], methods for linking expression to clinical outcomes [11], ChIP-on-chip or genome-wide association studies.

We restrict our commentary to a personal perspective on those network analyses invented by our group that can be run entirely on gene expression data independent of other data sources. A formal and more detailed description of each of these methods, including the statistical output, can be found in the literature cited.

## Why skeletal muscle?

Skeletal muscle is a relatively well-understood tissue. From an anatomical viewpoint it is formed from repeated structural subunits, built up hierarchically from cell to muscle fibre via sarcomeres and myofibrils. The development of muscle from embryonic precursor cell populations to mature adult organ is understood to be largely regulated by a mere handful of pro-myogenic transcription factors (*MYOD1MYOGMEF2CMYF5MYF6*) [12].

Skeletal muscle appears to us to be an appropriate, tractable tissue upon which to invent and calibrate new analytical and computational methods. The generality of the methods can be assessed by applying them more broadly to other species, tissues and circumstances and comparing the output against the experimentally validated literature. Unfortunately, there are not many cases where comprehensive gene expression contrasts have been performed in circumstances where a causal mutation has also unambiguously been identified.

## Gene expression data: promise and limitations

The last decade has witnessed the emergence of cheap, high-throughput techniques, including the microarray expression platforms. Microarrays, and the subsequent RNA sequencing based methods, provide detailed simultaneous expression information for tens of thousands of genes. The standard gene expression paper consists of lists of DE genes followed by enrichment analyses performed by web tools such as GOrilla [13], GSEA [14,15], DAVID [16] or Ingenuity Pathway Analysis (http://www.ingenuity.com/).

Informed commentators have suggested that gene expression technologies have failed to live up to their promise of providing molecular and systems-level explanations of biological phenomena, because the ability to extract meaning from the data is poorer than the ability to generate the data in the first place.

One of the more vocal critics, Sydney Brenner claims that inferring models of physiology and development from descriptions of molecular events in a complex system is an insoluble ‘*inverse problem*’ [17,18]. By way of analogy, Brenner argues that this is rather like inferring the physical properties of a musical instrument based entirely on a recording of the sounds emitted. Brenner argues that the approach is ‘…*bound to fail…*’ because the ‘*measurements taken are static snapshots*’ and that ‘…*information is lost.*’

Therefore, so the argument runs, while the data generation is enormous (“*high throughput*”) the gain in knowledge or understanding has been minimal (“*no output*”). Brenner makes a related claim that the natural functional unit in biology is the cell, and that consequently an understanding of biology rests on cell-level analyses [18].

Our overarching aim is to outline how gene expression data can be exploited to say something meaningful about the regulatory biology of skeletal muscle [1,2], and also how exactly the same methods have been applied to other (non – muscle) circumstances with success [4]. We will show that providing a reasonable resolution of the inverse problem of bovine muscle physiology and development can be achieved without recourse to the functional unit that is the cell.

The set of arguments that follow are built on a very simple insight, made independently by a number of groups (reviewed in [9]). To understand the regulatory *behaviour* of molecules in a complex system, they must not be considered in isolation, but rather in the context of other molecules.

Computing DE, the most commonplace first pass gene expression analysis, necessarily involves comparing a molecule to itself. Because this process neglects contextual information it is a form of inappropriate reductionism.

In trying to understand why a phenotype changes, one should not merely think “which gene(s) are the most differentially *expressed”*, but rather “which gene(s) are the most differentially *connected.”* This insight introduces us to the field of network science. Defining and exploiting differential connectivity has been an area of active debate and the details of the definition strongly influence its utility.

## The model system

We have studied *Longissimus* muscle in two breeds of cattle across development using the Bovine Agilent microarray platform [1,2]. One of the breeds, the Piedmontese, is extremely muscular due to a missense mutation which interferes with the function of the muscle growth repressing *MYOSTATIN (MSTN)* gene. Its counterpart, the Wagyu, possesses a wildtype *MSTN* gene and has a normal level of muscularity. Readers interested in the biological nature of *MSTN*, and how mutations in it govern mammalian muscle mass are directed to the following references [19-21].

Suffice to say, there is very little question that the muscularity of the Piedmontese breed can be largely attributed to a mutation in the *MSTN* coding sequence. The converging lines of evidence emerge from genetics [19,20], functional genomics [1], cell biology [22-24] and physiology [25,26].

Thus, we have a colossal amount of gene expression data on a well understood – albeit complex - tissue system, coupled with knowledge of both the physical site and functional *modus operandi* of a particular genomic mutation. This rich, numerical playground has afforded us an unparalleled Systems Biology opportunity to explore, discover and create new questions and solutions.

We have developed a series of universally applicable numerical analyses to correctly reverse-engineer core biological processes. Until now, these findings have been published in specialised technical journals, and explained in domain-specific mathematical language [1-4].

Given a well designed gene expression experiment, we can now identify with some confidence 1) the likely cause(s) of any perturbation (genomic or otherwise) and 2) how those cause(s) are communicated through the gene regulatory network. By good experimental design we refer to the usual statistical rules-of-thumb of adequate replication and randomisation. None of these are peculiarities of the particular methods discussed herein.

Heeding Einstein’s and Tukey’s heuristic advice, we have already made strides defining what the right questions are and so can now start giving meaningful, even if only approximate, answers. We believe we are now set for an era of “*well designed input, high throughput, carefully considered inferential algorithms, cogent output*!”.

## RNA data and meaning

During a gene expression experiment RNA is extracted from a tissue sample. One takes a tissue sample that is highly structured (comprising intact cells, organelles and so on) and thus from a biological perspective *information-rich*, and homogenises it to something unstructured and thus *information-poor*.

From an Information Theory point of view, the entropy, or disorder, has increased. Furthermore, the resulting RNA combines the various expression signals from the original sample, frozen at that point in time. The gene expression profiles reflect the cell-specific transcriptomes skewed by the overall cell composition of the sample.

The RNA samples are a ‘riddle wrapped up in an enigma’: highly complex in origin, highly disordered in preparation.

One of the main tasks facing the researcher is to try to ‘recover’ this starting information during the subsequent analyses. The answer lies in various iterations, some simplistic and some advanced, of the “*honour by association*” heuristic. In other words, we will show that much biological information can be recovered from global, systematic patterns of similarities and differences in the various expression profiles.

These systematic patterns involve not only comparing a molecule to itself, but also to other molecules. The higher-order analyses (Regulatory Impact Factor analyses) are built up from the ‘bricks’ of the more basic analyses (abundance, differential expression, co-expression and differential co-expression).

The mathematical formalism pertaining to each step is detailed in Tables 1 and 2. However, it is not necessary to follow the equations in order to appreciate the arguments.

**Table 1:** Measures of gene expression in ascending order of complexity

**Measure**	**Algebra formulae**	**Description**	**Example in skeletal muscle context**
Expression	$E_{i,A}=\frac{1}{n}\sum_{k=1}^{n}x_{i,k}$	Average (normalized) expression of the *i*-th gene across the *n* samples (eg. biological replicates) of experimental condition *A* and where each *x*_*i,k*_ corresponds to the expression of the *i*-th gene in the *k*-th sample (*k* = 1, …, *n*).	*MYL2* is abundant, *MSTN* is intermediate
Differential Expression	$dE_{i}=E_{i,A}-E_{i,B}$	Difference in the expression of the *i*-th gene in the two conditions under scrutiny, *A* and *B* (eg. healthy and diseased, two breeds, two diets, two time points, …). Note that it is not a requirement to have the same number of samples surveyed in the two conditions.	*MYL2* relatively strongly, definitely not *MSTN*
Co-Expression	$C_{i,j}=r_{A}\left(i,j\right)=\frac{Cov\left(i,j\right)}{\sigma_{i}\;\sigma_{j}}\;$	Similarity of expression profile (typically and shown here the Spearman correlation coefficient) between the *i*-th and the *j*-th genes across the *n* samples of condition *A*.	*MYOD1* and *MYOG*
Differential Co- Expression	$dC_{i,j}=r_{A}\left(i,j\right)-r_{B}\left(i,j\right)$	Difference in the co-expression between the *i*-th and the *j*-th genes in the two conditions under scrutiny, *A* and *B*. Note that it is not a requirement to have the same number of samples surveyed in the two conditions.	*MSTN* and *MYL2*
Co-Differential Expression	$CdE_{i,j}=r\left(dE_{i}\;\text{,}\;dE_{j}\right)$	Similarity of the profile of differential expression of genes *i* and *j* across the levels of another experimental design effect such as time points. Two conditions, *A* and *B*, are being surveyed across a series of developmental time points.	*MYL2* and *MYL3*

**Table 2:** **Higher-order metrics arising from combinations of the basic measures documented in Table**1

**Measure**	**Algebra formulae**	**Description**	**Example in skeletal muscle context**
Phenotype Impact Factor	PIFi=12Ei,A+Ei,BdEi=AidEi	Average (normalized) expression of the *i*-th gene across the two conditions multiplied by its differential expression. In other words, PIF weights the differential expression of a given gene by its overall abundance.	*MYL2* very strongly.
Regulatory Impact Factor, Option 1	$RIF1_{i}=\frac{1}{n_{\mathrm{dE}}}\;\sum_{j=1}^{j=n_{\mathrm{dE}}}\left(PIF_{j}dC_{i,j}^{2}\right)$	For the *i*-th regulator and across all the *j* differentially expressed genes (*j* = 1, …, *n*_*dE*_) RIF1 looks at the average PIF of the *i*-th regulator weighted by the squared differential co-expression between the *i*-th regulator and the *j*-th differentially expressed gene. It addresses the question: Which regulator is consistently highly differentially co-expressed with the abundant differentially expressed gene?	*MSTN* very strongly.
Regulatory Impact Factor, Option 2	$RIF2_{i}=\frac{1}{n_{\mathrm{dE}}}\;\sum_{j=1}^{j=n_{\mathrm{dE}}}\left[{\left(E_{j,A}r_{A}\left(i,j\right)\right)}^{2}-{\left(E_{j,B}r_{B}\left(i,j\right)\right)}^{2}\right]$	For the *i*-th regulator and across all the *j* differentially expressed genes (*j* = 1, …, *n*_*dE*_) RIF1 looks at the average change in predictive ability of the *i*-th regulator to predict the abundance of the *j*-th differentially expressed gene. It addresses the question: Which regulator has the most altered ability to predict the abundance of differentially expressed genes.	*MSTN* very strongly.

A small number of molecules will be used to illustrate the concepts, pre-eminently *MSTN*, the causal mutation in our particular (Piedmontese versus Wagyu) contrast. The essay is structured such that we begin with the simplest analyses and culminate with the most complex. At the conclusion of each contributing section it will be shown that *MSTN* cannot be identified, at which point we will proceed to a more complex and compelling approach.

The pros and cons of each analysis will be briefly discussed in passing, with specific illustrations given from our skeletal muscle data. The final section will reveal the nature of the differential connectivity analysis that can indeed quantify the long transcriptional shadow cast by *MSTN*.

## Expression (abundance)

The simplest expression information, taking each gene one at a time, is its abundance either in absolute terms or relative to other genes in the same experimental setting. The collection of mRNA’s in the sample can be viewed as a transcriptional ‘community.’

**Figure 1:**
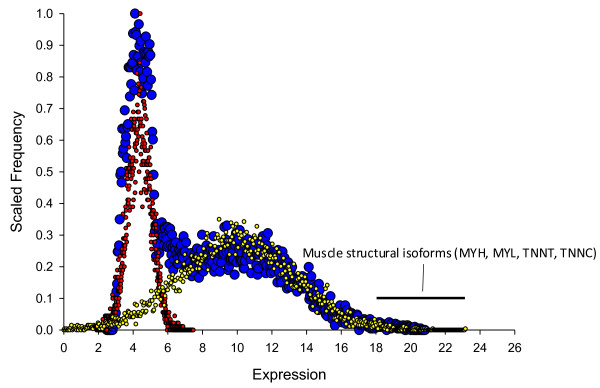
**Frequency histogram of bovine skeletal muscle transcripts (blue circles).** The overall distribution is bimodal (simulated by the red and yellow circles), and a relatively small number of highly abundant transcripts encoding muscle structural subunits, ribosomal proteins and mitochondrial proteins dominate. *MSTN* sits in the nexus between the two distributions, possessing an average abundance of ~7.

Examining muscle from a global perspective, this community is somewhat biased. That is, a relatively small number of molecules (the myosin heavy and light chain isoforms plus other major structural components such as ribosomal and mitochondrial proteins) dominate the sample, yielding a scale-free frequency distribution with a steep, exponential decay curve (Figure 1).

An examination of Figure 1 shows that the distribution in expression in skeletal muscle tends towards bimodality. Even after normalisation the shape is better described by a mixture of two partly superimposed normal distributions, rather than one normal distribution. Whether or not the shape of the distribution changes if the data are collected by microarray is not clear, and further refinement awaits direct RNA sequencing count data. However, the correlation between RNA sequencing reads and microarray probe intensities tends to be quite reasonable [27] so major changes seem unlikely.

Across tissues, the shape of these distribution curves can vary [28]. For example in a plant context, ‘leaves’ possess a biased transcriptional community whereas ‘pollen’ possess a more uniformly distributed transcriptional community [29]. It is not clear what these distributional differences mean, but one appealing hypothesis is that evenness reflects relative levels of ‘generalism.’ By this reasoning, the biased transcriptome of skeletal muscle reflects a dominant cell type (the myocyte) and its’ primary role of contraction. These issues of diversity and specialization have been systematically formalised for a range of human tissues by [30].

The enormous enrichment for the core muscle structural proteins among the very abundant muscle genes, suggests abundance gives an indication of the *structural contribution* a molecule makes to the sample. That is, molecules making a large contribution to tissue structure tend to be abundant at the mRNA level. A corollary is that molecules which encode structural proteins yield well to prioritisation based on this thinking.

However, as biologists interested in deciphering how and why a phenotype is formed, we are often more interested in the ‘input’ regulatory molecules as opposed to the ‘output’ structural molecules. But herein lies the problem: regulatory molecules are enigmatic, they tend not to conform to this reasoning i.e. the belief that their activity is closely related to their abundance.

**Figure 2:**
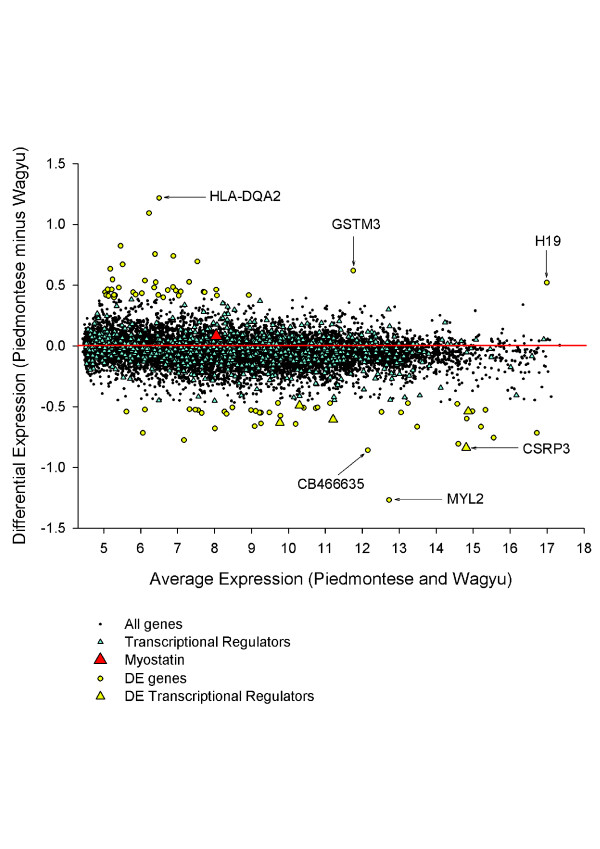
**Needle in a numerical haystack.** Despite being the causal effector molecule, *MSTN* is neither DE nor abundant when comparing *MSTN* mutant cattle versus *MSTN* wildtype cattle. Here DE is computed by subtracting the average expression in the Wagyu from the average in the Piedmontese, across the 10 time points. Figure from *PLoS Computational Biology*.

For example, in a genome-wide census of human transcription factors, Vaquerizas et al. [31] noted that regulators tended to be lowly expressed compared to other types of molecules. Equally, in our data we find that many key muscle regulators have low to moderate expression, including *MSTN* (Figure 2).

This means that one cannot determine *MSTN*’s importance to muscle biology through its expression level, which is modest. To return to the opening quotes, an accurate measure of abundance may well be the right answer (for some genes in some circumstances), but unfortunately for *MSTN* in this context it is to the wrong question.

This observation undermines any metric that is directly derived from abundance, such as differential expression.

## Differential Expression (DE)

From a statistical inference standpoint, the standard analysis is calculation of significant DE. This is the difference in the abundance of a given molecule in two treatments or conditions of interest. Typically, a table will be produced ranking the molecules in descending order of differential expression, and the list may be explored for functional enrichment using a burgeoning suite of readily available tools e.g. [13,16,32].

One drawback of these tools is that they provide biological interpretations based on what is already known, has been correctly entered into a database, and has adequate search and retrieval facilities. There is clearly a need for alternate analyses that can take the results from any experiment and make useful predictions and conclusions, irrespective of existing knowledge gaps.

Figure 2 shows the DE genes in our data in the form of a ‘MA’ plot (where ‘M’ and ‘A’ stand for ‘minus’ and ‘average’, respectively). Rare transcripts appear to be more DE, which is largely the result of poorer signal to noise as the detection limits of the technology are approached. Any ranking on DE, in the absence of sensitivity testing, will therefore have the unfortunate tendency of highlighting noise. A complication to this argument is provided by stochasticity. Namely that key developmental genes like members of the HOX family tend to be tightly expressed [33] while environmentally-responsive genes such as yeast detoxification genes change markedly in a variety of stressful conditions [34]. This stochasticity may provide a real biological source of variation independent of technical noise. Readers interested in further discussion on robustness versus stochasticity are directed to this review [35].

Putting stochasticity to one side, sensitivity testing [36] broadly helps overcome this problem, by requiring a greater DE for lowly expressed genes to be called significant. In a complementary approach, we have invented an adapted differential expression metric called Phenotypic Impact Factor (PIF). This is the product of a molecule’s DE and its average abundance [1,4].

This statistic has two desirable features. Firstly, DE and abundance correspond to the two sources of variation in the original data. As they are independent of each other, their product accounts for all the variation, so the computation of the metric does not lose valuable information. Secondly, weighing by abundance reduces the effect of noise in the rarer transcripts, and increases the ability to detect small changes in the abundant ones. This process highlights molecules making an important structural contribution to the *change in phenotype,* forming the foundation of downstream analyses.

For example, in our data, ranking on PIF rather than DE does a much better job of emphasising the slow myosin isoforms driving the change in fibre composition that accompanies the *MSTN* mutant phenotype [1].

**Figure 3:**
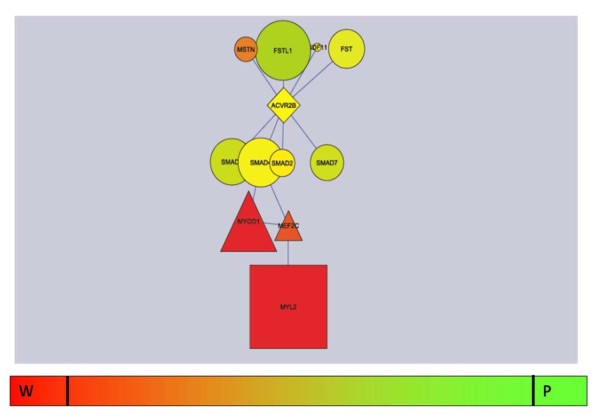
**Comparing the*MSTN*muscle building pathway in the two breeds.** The yellow (no differential expression), red (upregulated in Wagyu) and green (upregulated in Piedmontese) colours were generated within Cytoscape, with the bright red and bright green representing the outermost bounds of the extreme DE molecules across the whole transcriptome at day 135 post conception. The colour bar beneath the molecular pathway is intended as a schematic guide only. ACVR2B was not reported on the array, but was included in the diagram for visual completeness of the pathway.

There are a number of different ways of computing DE in longitudinal data (comparing single time points, or various combinations of time points). In our data, whichever DE measure is used, *MSTN*, the causal regulator, is not prioritised. This is illustrated in Figure 2 for all time points averaged, and in Figure 3 for a single particularly important developmental time point only, that of secondary myogenesis. We have also computed DE at every other time point taken individually and can confirm that *MSTN* is not significantly DE at any of them. Therefore, in a concerning false negative, *MSTN* will be overlooked by any analysis using DE as the exclusive prioritisation strategy. An accurate measure of DE is again the right answer to the wrong question.

We wish to emphasise that this observation is much more than an isolated curiosity. The same faulty reliance on DE applies much more broadly, interfering with and confounding our comprehension of the entire regulatory landscape.

We know that there are a large number of constitutively expressed regulatory molecules whose activity is known to be modulated almost entirely post-transcriptionally i.e.. via reversible phosphorylation, cellular localisation, cofactor or ligand binding and so on. These changes in activity are entirely invisible to DE analysis as they relate, in various guises, to a molecule’s *behaviour* not its own *abundance*.

From a biological knowledge perspective, this may seem a statement of the obvious. However, from an analytical perspective, if we rely on DE we are neglecting this appreciation. We shall illustrate the pervasiveness of this problem with a few examples from the very recent literature. On the 21^st^ November 2011 one of us (NJH) searched for “microarray gene expression” on NCBI Pubmed. The 3 most recent hits were as follows [37-39].

One [39] describes a new statistical approach to document the role a cellular pathway plays in a change in phenotype (in this case, cancer versus normal). The authors describe their method as follows “…a larger value indicating relatively higher expression levels of genes in a pathway and therefore a higher pathway activity.”

A second example [37] uses gene expression to contrast Duchenne Muscular Dystrophy subjects with unaffected control subjects. In the abstract the authors write “We identified 528 differentially expressed genes, of which 328 could be validated by an exhaustive meta-analysis…”

In the third example [38], the authors used microarray gene expression to contrast bovine ileum response to pathogens versus control animals. They write “gene expression changes mapped to 219 molecular interaction pathways and 1620 gene ontology groups.” Furthermore, “…a period of intense gene expression activity…primarily increased gene expression at later time points…consistent between microarray and qRT-PCR for both up-regulated and down-regulated genes.”

Furthermore, a very recent review [40] states that gene set enrichment analyses are “…commonly applied to identify enrichment of biological functional categories in sets of ranked differentially expressed genes from genome-wide mRNA expression data sets.”

Abundance, abundance, abundance! So, how does one get a measure of a molecule’s behaviour?

## Networks and ‘contextomies’

Computation of DE is a form of reductionism that overlooks molecular interaction or context. According to Wikipedia, a ‘contextomy’ is a type of false attribution in which a text passage is removed from its surrounding material in such a way that its meaning is distorted. We believe that computing an isolated list of DE genes is analogous to performing an accidental ‘molecular contextomy.’

What is required to identify causal molecules like *MSTN* is a clear sense of their *molecular context*. The branch of mathematics that deals with this area is network science. The remainder of this essay will deal with the application of clustering-based approaches to infer networks from expression data.

This problem in the specific case of our data is made most apparent in Figure 3, illustrating not only the absence of DE of *MSTN* itself but also that of the particular muscle-building component of the TGF-β signalling pathway into which *MSTN* is embedded [41]. Even though we are looking in the right place (a sub section of the TGF-β pathway) at the right time (secondary myogenesis), neither *MSTN* nor any of the associated up nor downstream signalling or regulatory pathways are DE at this time point!

What is happening? In short, the impact of the perturbed TGF-β pathway is entirely post-transcriptional. That is, in the muscular Piedmontese the mutated *MSTN* fails to bind the activin IIB receptor *regardless of its own expression level*; *SMAD* complex formation and nuclear translocation fails to occur *regardless of their own expression level* – each successive step passing on the TGF-β pathway information independent of expression level.

While this will not be a surprise to practicing biologists, the set of commonly applied gene expression statistics reliant on DE, in effect, ignore it.

In our data it is only the change in expression of the pro-myogenic TF (*MYOD1*) and the other output or target genes (such as *MYL2*) at the very end of the chain that can be perceived by DE. But even knowing the nature of the pathway *a priori* as illustrated in Figure 3, this output information is still inadequate to find a causal role for TGF-β. There is nothing in the DE data that connects *MSTN* to *MYL2*, because none of the intermediates are DE either.

Admittedly, Figure 3 is a convenient schematic to illustrate the point, derived from the orthologous pathway in mouse [41]. It may be incomplete. However, future refinements to the pathway and the inclusion of more component parts will only confuse matters more, unless a previously unrecognised TGF-β muscle building component turns out to be highly DE, or RNA sequencing count data reports as highly DE a TGF-β molecule not reported at all by the Agilent array technology. Even accepting this (perhaps unlikely) scenario, any conclusion about the possible role of *MSTN* would still be under a cloud because of the lack of DE in all the other components. Our full data set can be found in [42] for readers interested in exploring the data more thoroughly.

We wish to emphasise that this observation says nothing about technological limitations. It is a much stronger ‘in principle’ argument. *Using DE alone* it is theoretically impossible to determine that *MSTN* is doing anything interesting in these data. However excellent ones measure of *MSTN*s abundance, one will not see anything because abundance is not what changes. For example, one might replace microarray probe data with more sensitive qRT-PCR data. However, any increase in sensitivity achieved for detecting the DE of *MSTN* would presumably be matched for all the other genes in the system. Consequently, this more sensitive approach would not actually help with the correct prioritisation, because there is no reason to believe the rank order would change appreciably.

This observation is devastating for the burgeoning suite of tools that are routinely exploited by biologists to identify serial components of a causal pathway from isolated DE gene lists, such as Ingenuity Systems Pathway Analysis (http://www.ingenuity.com), GOseq [32], DAVID [16], GOrilla [13] and many, many more. It should be pointed out that these web tools can utilise any form of ranked gene list as input, and are not reliant on those derived from DE, even though DE lists are a common practical choice. Therefore, our main point is a criticism on the exclusive use of DE lists as input for the tools, rather than the use of the tools *per se*.

Up until now, we have discussed a set of approaches that do not work in the data under consideration. In the network analyses that follow, genes are grouped based on some measure of profile similarity - the “*honour by association*” heuristic. The more sophisticated the analysis, the more penetrating the question, and the deeper they dig into the data. We shall begin with the most basic and familiar, “co-expression.” And we shall end with the most complex, “co-differential co-expression.”

The initial analyses are able to identify certain known regulatory relationships. However, they miss a lot, including the role of *MSTN*. The final analyses can correctly identify not only *MSTN* as the likely causal mutation, but also the regulatory molecules to which it is communicating when mutated.

## Co-expression

**Figure 4:**
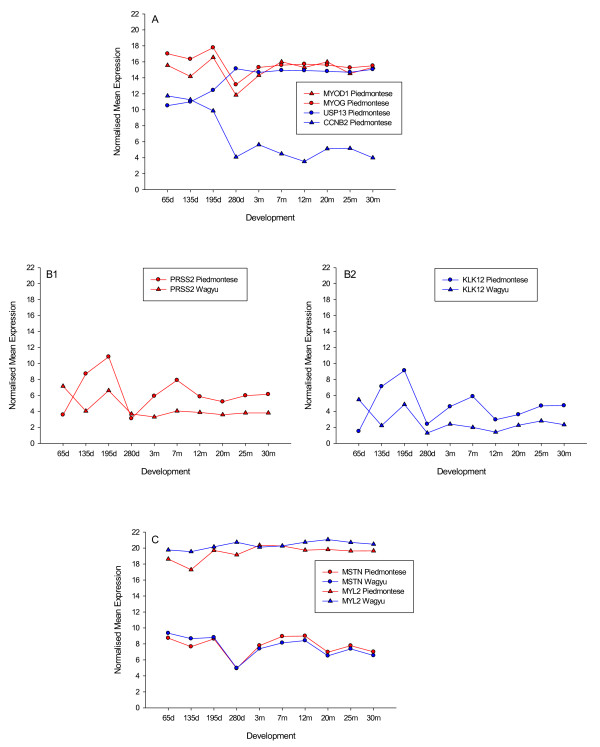
**Co-expression, co-differential expression and differential co-expression.** As illustrated for the Piedmontese across development, *MYOD1* and *MYOG* are strongly positively *co-expressed* whereas *USP13* and *CCNB2* are strongly negatively co-expressed **(A)**. In comparing Piedmontese and Wagyu, *PRSS2* and *KLK12* are co-ordinately or *co-differentially expressed* in addition to being co-expressed **(B)**. The *differential co-expression* arrangement between *MSTN* and *MYL2* is large (+1.1), despite the co-expression strength being relatively modest in the Piedmontese (+0.76) and Wagyu (−0.34) breeds treated separately **(C)**.

This approach assesses the similarity in expression profile of gene pairs across various experimental conditions, based on a distance measure such as the Pearson’s correlation coefficient. In our data the expression profile of *MYOD1* is significantly co-expressed with *MYOG* in both Piedmontese and Wagyu animals across the ten time points (Figure 4A). Significant co-expression can be used as a criterion to build a co-expression network.

To determine co-expression significance we use Partial Correlation Information Theory (PCIT) [3]. It is our judge, jury and executioner. However, there are a number of alternate approaches in the literature [43-49].

PCIT is a soft-thresholding method that exploits the twin concepts of Partial Correlation and Mutual Information. In brief, it explores relationships between all possible triplets of genes, in an attempt to determine truly informative correlations between gene pairs *once the numerical influence of other genes in the system has been accounted for*.

The scale of this exploration is very large. For example, expression measurements for a typical microarray experiment of ~10,000 genes yield (10,000 × 9,999/2) ~50 million possible pair-wise correlations and (10,000 × 9,999 × 9,998/6) ~ 167 billion trios. Clearly, this presents potential scalability issues for higher resolution transcriptome data sets looking to the future, which are as yet unresolved.

This systematic and exhaustive exploration yields a global estimate of the numerical structure of a given data set, providing the broader context into which any particular pair-wise correlation must be assessed. This awareness of context means PCIT can be justly applied to data sets that possess, at a global level, varying strengths of inter-relationships.

Depending on the particular data set, a ‘weak’ correlation of 0.6 may in fact be deemed significant (if the correlation is mainly attributable to the two genes in question and not an artifact of any of the genes in the pair being highly correlated to a third gene in the dataset), whereas a ‘strong’ correlation of 0.9 may be rejected (if it is mainly attributable to the third member of the triplet).

What does a co-expression network look like? Our bovine muscle network possesses several interesting properties [2]. Firstly, it has a scale-free Power Law connectivity distribution; a few highly connected nodes or ‘hubs’, with many poorly connected nodes. This non-random connectivity distribution is commonly observed in many types of real-world networks.

Secondly, in contrast to ‘small-world’ networks, such as the 6 metaphorical handshakes that connect everyone on the planet (the Kevin Bacon Principal), it can take a surprisingly large number of steps to traverse a co-expression network.

In a real Gene Regulatory Network based on direct experimental observations, a highly connected hub would correctly be seen as a highly important regulator. Its targeted removal would have functional consequences. Indeed, one known feature of Network Theory is that scale free topologies are robust to random attacks and vulnerable to targeted attacks [50]. However, in a co-expression network context this seductive notion is an illusion.

In our network, *MSTN* is not connected to anything while *MYOD1* and *MYOG* are poorly connected. We conclude that in co-expression networks well-connected hubs are better seen as components of biological *structures* (such as muscle structural isoforms) and *processes* (such as the cell cycle) which themselves are tightly regulated, not quite the same thing.

A major take home message is that co-expression networks tend to resolve into highly inter-connected functionally-related ‘modules’ such as mitochondria, extracellular matrix and ribosomal proteins [2,51]. Although these were present as coherent structures in the original tissue sample, they were subsequently annihilated during RNA extraction.

Therefore, when visualising co-expression networks such as our bovine muscle network, one recovers a great deal of structural and functional information that was *apparently* lost during sample preparation – offering support for clustering-based methods [2].

An additional appealing feature of these networks is that by analysing entire modules of genes, rather than single genes, one is able to interpret relatively small changes. For example, one can overlay the DE results of a separate experiment on top of the co-expression network [52]. Observing an entire module of highly co-expressed genes, each represented by an independent probe, all increasing a mere 1.1 fold adds confidence to this small change. This reasoning helps overcome the sensitivity issues levelled at microarrays – notwithstanding our criticism of thinking solely in terms of DE.

Furthermore, high co-expression is reasonably successful in allowing real regulator-to-target interactions to be determined [2]. For example, by hunting in the module of interest or asking the question “*which regulator has the highest absolute average correlation to all the genes in the module?*” one correctly infers driver of core processes such as cell cycle (*E2F1*), mitochondrial biogenesis (*ESRRA*) and muscle fibre composition (*SIX1*) [2].

Nevertheless, the co-expression approach tends to select the low hanging fruit. *MSTN* is absent from our co-expression network; it is not significantly co-expressed in both breeds with any gene. An accurate measure of *MSTN*’s co-expression significance is once again the right answer to the wrong question.

## Co-differential expression

Co-differential expression clusters together groups of genes whose profiles may not necessarily be co-expressed, but they are differentially expressed in a coordinated fashion; even though they are not similar, they are *similarly different*. In our case, the patterns of differential expression are coordinated over time (Figure 4B).

In principle, co-differential expression can be used to build networks, although we cannot find a precedent for this in the literature.

This property of relatively strong co-differential expression but relatively weak co-expression is true in our data for a broad set of slow twitch muscle fibre structural proteins [1,2].

In essence, one is computing a ‘shape-based’ version of DE. This is defined by the surfaces described by the profiles of various genes in two different conditions. Co-differential expression is a useful feature for connecting genes that might be missed by conventional co-expression. As with co-expression, the linking makes a prediction about shared biology e.g. peptidase activity in the case of *KLK12* and *PRSS2* (Figure 4 panels B1 and 2). In the example given, *KLK12* and *PRSS2* are co-expressed in addition to be co-differentially expressed, although this need not always be the case.

## Differential co-expression

“What is the question to which MSTN is the answer*?”*

Transcription Factors and associated molecules are often considered to be the hubs in gene regulatory networks. It is known that they tend to be stably expressed at relatively low levels. Their activity is controlled largely at the post-transcriptional level, by phenomena such as nuclear translocation of the protein, binding of activating co-factors or ligands and so forth. This means their own expression level is a poor measure of how active they are in a given biological situation.

As mentioned previously, a corollary of this is that DE will not be an appropriate measure of a change in their activity, and subsequently will often fail to prioritise them correctly. Equivalent arguments also apply beyond TF to other regulatory molecules, such as components of signalling pathways. Based on these considerations we believe DE analysis will lead to poor prioritisation of causal regulatory pathways in a host of species, tissues and biological circumstances, although it may perform well with close-to-target output pathways.

Returning to our specific example, *MSTN* is neither 1) abundant nor 2) differentially expressed nor is it 3) consistently significantly co-expressed in Piedmontese versus wildtype Wagyu muscle. It is the proverbial needle in a (numerical) haystack. Nevertheless, in a retrospective analysis trying to determine “*the biologically sensible question to which MSTN is the answer*” we did discover the smoking gun [1].

**Figure 5:**
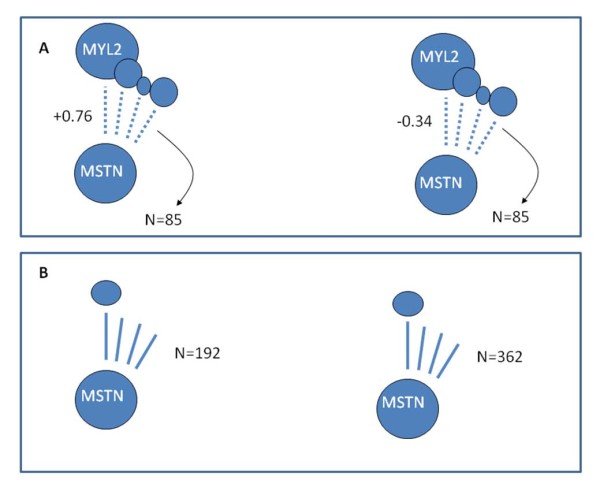
***MSTN*is highly differentially co-expressed with many of the abundant, highly differentially expressed genes - mutant breed on the left, wildtype breed on the right.** For example, *MSTN* has a differential co-expression of 1.1 (+0.76 - - 0.34) with *MYL2* (Panel **A**). RIF accumulates these differential co-expressions for all the DE genes (85 in this instance), weighted by their abundance. The size of the bubble representing the various DE genes corresponds to the combination of the extent of DE and average abundance. An alternative measure of differential connectivity is given in Panel **B**, where the number of significant co-expressions possessed by *MSTN* in the two breeds is contrasted. *MSTN* does not get prioritised by this alternative approach.

In brief, *MSTN* is strongly differentially co-expressed to many of the 85 DE genes, some of which are highly abundant and highly DE. The DE genes represent putative ‘targets’ for the regulatory molecules, and the word ‘target’ will be used in this rather relaxed manner throughout the rest of the correspondence. These differential co-expression relationships are presented schematically in the top panel of Figure 5. MYL2 is highlighted by name on Figure 5 and in the discussion that follows because it best reflects the combination of high abundance and high differential expression which the RIF algorithm exploits when weighing the differential co-expression relationships.

For illustrating the principle under consideration, the discussion that follows will focus on the specific differential co-expression relationship between *MSTN* and *MYL2*. However, for the purposes of computing RIF, the DE profile of *MYL2* is just one contributor. In fact, *MSTN* is also differentially co-expressed with many other transcripts that are highly abundant and DE, and is best exemplified by those encoding muscle structural proteins: *ACTN2* (differential co-expression to *MSTN* of 0.59), *TNNT1* (0.45)*, MYOZ2* (0.43) *and MYL3* (0.30).

It is the combined weight of influence of these molecules that drives the gross anatomical change in fibre composition. Similarly, along with the other DE genes, the expression profiles of all these molecules contribute substantially (but to somewhat varying extents) to the RIF output. Thus, it is the overall weight of *MSTN*’s system-wide differential connectivity, not the behaviour of its relationship with any one DE gene, which permits its correct prioritisation.

In the wildtype Wagyu breed, across the ten time points, *MSTN* has a fairly weak negative co-expression arrangement with *MYL2* (−0.34). This negative co-expression reflects *MSTN*’s function as a negative regulator of muscle mass under normal circumstances. Thus, when *MSTN* goes up, muscle structural proteins like *MYL2* go down and *vice versa*.

However, in the mutant Piedmontese breed, the normal repression is lost because of the mis-folding of the mutated *MSTN* protein. The new – now modest to strong - positive co-expression arrangement (+0.76) reflects this change in protein behaviour. That is, when *MSTN* goes up, *MYL2* also goes up and *vice versa*. The differential co-expression arrangement is simply the difference in co-expression between the two breeds i.e. +0.76 – –0.34 = + 1.1. The maximum differential connectivity by this differential co-expression definition is 2 (i.e +1 – –1) and + 1.1 can be seen as very substantial, even though the co-expression values in the two breeds could be seen as somewhat modest when taken in isolation.

Because substantial changes to modest co-expression relationships provide such useful re-wiring information, no attempt is made during RIF to assess the significance of the individual (breed level) co-expression arrangements. That is, it is of no concern that the *MSTN-MYL2* co-expression is a meagre −0.34 in one of the states (i.e. breeds).

This contrasts starkly with formal comparisons between co-expression networks. Co-expression networks clearly rely on some assessment of co-expression significance, and most methods would likely dismiss this *MSTN-MYL2* relationship as non-significant.

Similarly, no attempt is made to place formal levels of significance on the various differential co-expression relationships. Nevertheless, it is true that small differential co-expression arrangements make a smaller contribution to the RIF output than the large co-expression changes, assuming the abundance and differential expression of the target gene remains constant. These differential co-expression arrangements are computed system-wide for all regulators (~1000) versus all DE genes (85 in our data).

This set of phenotypically relevant differential co-expression observations brings us much closer to the right question and therefore the right answer. The analysis works by assessing which regulators change their behaviour with the highly abundant, highly differentially expressed genes – which in turn reflect the *change in phenotype* at a molecular level.

These simultaneous relational properties can be accumulated into a single metric, which we call RIF [1]. Verbally, this can be expressed as “which regulator possesses the greatest total amount of differential co-expression to the very abundant, highly differentially expressed genes.” The numerical answer represents a holistic global differential connectivity statistic. The algorithm correctly identifies causal regulators in a range of data sets, irrespective of numerical structure, nature of the perturbation, tissue or species [4]. Unlike many network inference approaches, the RIF algorithm is computationally inexpensive. Consequently, we do not foresee scalability problems with higher resolution data sets into the future.

We have applied RIF to both breast cancer and colorectal cancer expression data sets, contrasting them against normal tissue samples. The breast cancer RIF analysis underscored the role of estrogen signalling [4], while the colorectal cancer analysis prioritised CDK8 [53], a known biomarker of the disease [54]. Furthermore, we note that an independent group has used Global Differential Wiring Analysis to identify a causal regulator of Parkinson’s disease (alpha syn-nuclein) based on brain sample gene expression. The Parkinson’s research output is currently Pending as a Patent [55]. None of these various discoveries could have been made by exclusive analysis of DE.

A summary of RIF results in a broad range of circumstances can be found in Table 3.The legitimacy of the prioritisations are repeatedly borne out by literature searches. However, in many gene expression data sets unequivocally defined causal mutations or effector molecules are not known, clearly making retrospective assessment more challenging than the particular *MSTN* case upon which the algorithm was first developed.

**Table 3:** Causal genes correctly highlighted by RIF across a range of species and biological circumstances

**Gene**	**Species, Phenotype**	**Independent evidence for gene function**	**RIF ranking**	**Differentially expressed**
MSTN	Cattle muscle, Piedmontese hyper-muscularity versus normal	Mapping, deep sequencing [60]	1^st^ out of 920 [1]	No
Alpha-Synuclein	Human brain, Parkinson’s disease versus healthy	Range of evidence including GWAS reviewed in [61]	Not formally stated in patent [55], presumably 1^st^	Unknown. Patent was established to identify causal variants by transcriptome wiring, even when not DE
CDK8	Human colon, colorectal cancer versus healthy	Colorectal cancer oncogene regulates B-catenin [54]	4^th^ out of 1,292 [53]	No
P107	Human, brown fat tissue versus white fat tissue	P107 knockout mouse exhibits a uniform white to brown fat transition [62]	5^th^ out of 552 [4]	No
DLK1	Sheep muscle, Callipyge hyper- muscularity versus normal	Not proven, but DLK1 is the most DE highly abundant gene, and its expression is maintained post-natally in effected muscles only.	4^th^ out of 898 Unpublished data	Yes, 2.14-fold up- regulation in callipyge individuals across all time points explored.
INSM1	Pig, 6 CNS tissues versus 21 other tissues	Neuroendocrine differentiation [63]	1^st^ out of 1,072 (submitted)	Yes, 3.8-Fold up- regulation in CNS
OXTR	Cattle muscle, steroid hormone induced muscling	No direct evidence, but OXT precursor is the most DE gene in this experiment, and is known to drive cardiac development.	2^nd^ out of 2,944 [52]	No
CARM1	Human breast, breast cancer high survival versus low survival	Regulates estrogen stimulated breast cancer via E2F1 [64]	2^nd^ out of 892 [4]	No

RIF highlights the correct molecules irrespective of whether they are DE.

By way of analogy, the RIF approach is similar to inferring where a stone has dropped into a pond by the surface disturbance. Working back from the emanating ripples (the output DE genes and pathways), allows one to identify the original splash (the input regulators).

It can be seen that the ‘pond algorithm’ transcends the detailed nature of both the object and the fluid. It works so long as the pond is visualised at the appropriate time points, otherwise the signal will not be detected. If one looks too early the ripples will not yet have been produced, and if one looks too late the surface will have re-equalized.

In the same way that one does not need to see where the stone drops, nor worry about the exact physical nature of the stone or pond, one does not need the causal regulator(s) to be DE, nor does one need to know the exact biological details of the rewired molecule(s).

Assuming a set of gene expression samples are collected at the relevant time points, the approach will universally work.

The algorithm operates at a deep level of abstraction, by proxy rather than directly. Returning to Figure 4 (panel C) and Figure 5 (panel A), it is the differential co-expression between *MSTN* and *MYL2* (and the other abundant, DE genes such as other fibre specific subunits) that supplies the analytical leverage. From a certain pragmatic perspective, the fact *MYL2* is not a direct target of *MSTN* does not interfere with the correct prioritisation of *MSTN* even though this detail is clearly of biological relevance. In our approach the identification of the ‘targets’ is entirely data driven by DE, and is a proxy of transcriptional disturbance.

Does it matter that the DE targets may not be immediate, direct targets of the regulators under consideration? This question is related to the problem of inferring causation from correlation. It also returns us to Brenner’s original criticisms, and introduces us to the topic of emergence in complex systems science.

In the physical and chemical sciences, the robust quantitative Laws and Models possess a common thread. They are emergent Laws built on emergent data.

There are very few, if any, truly foundational Laws. By foundational, we mean those describing processes at lower levels of organisation that can correctly predict processes at higher levels of organisation. For example, and perhaps contrary to naïve expectations, the very reliable ‘macro-scale’ Laws of Thermodynamics accurately model emergent statistical properties that are actually simpler than the complex ‘micro-scale’ interactions upon which they are based.

How does this physical modelling discussion relate to gene regulatory network inference? In our complex (developing tissue) system, the real impact of the *MSTN* mutation is manifest at multiple biological scales: micro- (impaired binding of *MSTN* to the *MSTN* receptor *ACVRIIB*[56] at the *level of the molecule*), intermediate- (extended cell proliferation at the *level of the cell*) and macro- (gross change in muscle fibre composition and muscle mass at the *level of the tissue*). These smaller scales are very complex. They depend on molecular binding affinities, sub-cellular and extra-cellular molecular movement, and exquisitely coordinated patterns of cellular dynamics.

However, we wish to point out that the transcriptional outputs we measure (and base our network model on), integrate from and thereby ‘emerge’ from those various scales – *even if we don’t address any of them explicitly*. From this conceptually abstract perspective, it is irrelevant that *MSTN* does not directly activate or repress *MYL2.* For the purpose of computing RIF*,* all that matters is that these various multi-scale events set in motion by the *MSTN* mutation are reflected by a measurable transcriptional change in output DE genes like *MYL2*.

In terms of biological inference, the RIF output implies *MSTN* is involved at some level in driving the difference in expression of *MYL2*, even if there are considered to be intermediate molecular steps which hitherto remain elusive. That being said, the next section will discuss how the RIF output can subsequently be wired into a very plausible gene regulatory network linking together some of the expected intermediates (including MyoD1) that sit between *MSTN* at the beginning of the chain, and molecules like *MYL2* at the end.

Furthermore, no attempt has been made to try to unravel the component cell-specific profiles. In this instance it transpires that one does not need to explicitly think in terms of the cell, in order to infer something meaningful about the overall tissue.

What does this mean, in light of Brenner’s arguments? To summarise, we would reply that 1) high throughput expression measurements can be *accurate enough* for global questions; 2) an explicit account of the cell is not necessarily required for meaningful inference; 3) if one reduces *‘in context’* one does not necessarily lose important information; and 4) inverse problems can be tractable if your technology provides emergent data upon which emergent models can be built.

While Brenner’s musical instrument argument may be logically correct in that specific instance (i.e. his conclusion follows from his premise), it appears to us that the analogy is not a good one. Brenner’s negative conclusion does not translate well to gene expression analysis.

When applying RIF to these data, one boils down from 10,000 genes, two breeds and 10 time points to a single statistic, ranking *MSTN* first. Clearly, this is an act of reductionism, but because it reduces ‘in context’ it is holistic in spirit.

## Co-differential co-expression

We have seen that one can identify *MSTN* as the likely causal effector through global patterns of differential co-expression. A related question is: which molecules is the mutant *MSTN* communicating with in Piedmontese cattle to generate the muscular phenotype? In other words, can we build a re-wired ‘differential gene regulatory network?’ We have found this can be determined by asking which molecules possess an equivalent pattern of differential co-expression to the abundant differentially expressed genes.

To pursue the pond analogy, this is rather like simultaneously examining the pond for similar looking patterns of emanating ripples (in different locations on the ponds surface), then inferring something in common between the objects causing those patterns. In our data, applying the PCIT significance testing algorithm to the various differential co-expression relationships produces a ‘co-differential co-expression network’ [1]. Other than our own work [1], there is no precedent for this type of network in the literature. It can be contrasted with the more traditional application of significance testing approaches (such as PCIT) to expression profiles, which produce co-expression networks.

This more sophisticated ‘differential network’ establishes a link between *MSTN* and just two other regulatory molecules - *MYOD1* and *IFRD1*. Satisfactorily, in mice, *MSTN* is known to form an *in vivo* regulatory circuit with the orthologs of both these molecules [57,58]. This close inter-relationship between *MSTNMYOD1* and *IFRD1* cannot be determined by simple co-expression analysis, and provides compelling but not definitive proof for the soundness of the concept.

Compared to a co-expression network, the co-differential co-expression network has an unusual global topology. Rather than resolving into several modules with a scale-free connectivity distribution, the vast majority of molecules are highly connected together into a single dense, cohesive network. In effect, they exhibit a similar absence of differential co-expression, implying they are similarly irrelevant to the change in muscularity. They create no ripples in the system.

Good examples of molecules in this category include PTTG1 and TOPO2A. They possess a very extreme near perfect co-differential co-expression of 0.994. They are both involved in the highly fundamental biological process of chromatid separation during DNA replication, which plays no particular role in driving relatively superficial processes like changes in muscle mass. This result, and many others like it, reinforces the power of clustering-based approaches to infer functional similarity.

On the other hand *MSTN*, *IFRD1* and *MYOD1* are an example of a small, isolated and independent sub-network. In contrast to the majority of the molecules in the system, they possess similarly high and coordinate levels of differential co-expression compared to the abundant DE genes.

We find it very appealing that a single clustering method (co-differential co-expression) can correctly connect those molecules that leave a big splash and apparently drive a phenotype change (*MSTN* plus connectors) and, simultaneously, those that leave no imprint and therefore are predicted to play little part in driving the change (*PTTG1* and *TOPO2A*). Standard co-expression can recreate the second of these, but not the first.

At this point, we wish to reinforce the statement that to the best of knowledge our understanding of Piedmontese muscling due to the combined re-wired activity of *MSTN, MYOD1* and *IFRD1* cannot be deduced from other biological approaches, whether analytical or experimental in nature.

The result cannot be deduced from DE because the muscle building component of the TGF-β pathway is not substantially DE. This strongly implies that no matter how well curated the molecular pathway is - or becomes - overlaying DE measurements onto it will not work, now or in the future. More sensitive measurements of abundance, such as qPCR or indeed future technologies are of no relevance here. They will systematically increase the sensitivity with which all genes can be deemed DE, and therefore make no appreciable difference to the rank order and by extension, the ability to prioritise the correct molecules.

As far as we are aware the correct prioritisation cannot be deduced from alternative differential connectivity algorithms. For example, we ran the *DiffK* differential network algorithm of [59] and it failed to identify *MSTN*[1].

Our attempts to contrast the number of significant (i.e. tight or highly coordinated) co-expressions in key molecules between the two breeds were also futile [1] (Figure 5 panel B). Along these lines, we feel strongly that contrasting ‘non-significant’ co-expression relationships is very important in differential network approaches. Ignoring large changes in modest relationships omits valuable re-wiring information. In this specific case, contrasting significant co-expressions produces a non-extreme differential connectivity result for *MSTN* (Figure 5 panel B)*,* presumably because it struggles to handle changes exemplified by the *MSTNMYL2* co-relationship.

The result cannot be deduced from DNA precipitation experimental methods because *MSTN* does not bind DNA. This also means that examination of Transcription Factor Binding Sites based on DNA sequence analysis are of little value here.

The causal role for *MSTN* could in principle be homed in on through GWAS assuming a marker proximate to *MSTN*’s chromosomal location is present on the chip. However, the regulatory circuit the mutated *MSTN* forms with *MYOD1* and *IFRD1* cannot be deduced from GWAS data by existing statistical methods unless all 3-way epistatic interactions are exhaustively searched, and certainly not if the change in behaviour of *MYOD1* and *IFRD1* is solely dependent on the specific genetic change in MSTN.

The only method we are aware of capable of inferring such a model of re-wired transcriptional regulators is a well-designed gene expression experiment followed by RIF and co-differential co-expression.

The appeal of our method can be found in two qualities. Firstly, it is entirely data-driven, requiring nothing else but transcriptome data. Secondly, it is very simple compared to the data it models so concerns of over-fitting have no foundation. Nevertheless, we welcome further attempts by other scientists to apply their preferred analytic methods to this expression data set, or indeed to use it to invent new approaches. Clearly, it is conceivable that complementary insights might be achieved by as yet undiscovered numerical strategies.

## Conclusion

In this essay, we have tried to show how it is possible to dig deep enough to uncover causal mutations and other causal effector molecules from gene expression data. As with all complex problems, the starting point, as agreed by Einstein, Tukey and others, is asking the right question.

Through RIF (*MSTN*) and ‘co-differential co-expression’ (*MYOD1, IFRD1*) one can make substantial progress in solving the Inverse Problem of Piedmontese enhanced muscularity using nothing more than the output behaviour of a complex system.

We have illustrated why “*which molecule is the most differentially expressed*” is not necessarily the right question. Any downstream interpretation built exclusively on DE, no matter how sophisticated, runs the risk of producing faulty scientific insights.

## Competing interest

The authors declare that they have no competing interests.

## Authors’ contributions

NJH, BPD and AR conceived of the commentary. AR performed the mathematical formalism. NJH drafted the manuscript. All authors contributed to, read and approved the final manuscript.
